# Crossing borders, facing barriers: a rights-based narrative review of HIV care access among African migrants living with HIV

**DOI:** 10.3389/fpubh.2026.1706232

**Published:** 2026-08-27

**Authors:** Joanne Ellen Mantell, Tsitsi B. Masvawure, Noelle Anna Thomas, Yael Hirsch-Moverman, Gavin George

**Affiliations:** 1 Department of Psychiatry, Columbia University Irving Medical Center, New York, NY, United States; 2 Department of Integrative and Global Studies, The Global School, Worcester Polytechnical Institute, Worcester, MA, United States; 3 Bioethics Program, Columbia University School of Professional Studies, New York, NY, United States; 4 Department of Epidemiology and ICAP, Mailman School of Public Health, Columbia University, New York, NY, United States; 5 Health Economics and HIV and AIDS Research Division (HEARD), University of KwaZulu-Natal, Durban, South Africa; 6 Division of Social Medicine and Global Health, Lund University, Lund, Sweden

**Keywords:** Africa, ethics, health equity, healthcare, HIV, human rights, migrants, structural violence

## Abstract

International migration has long shaped economies and public health. In today’s globalized world, migrant health is increasingly a critical equity issue. This narrative review focuses on healthcare access for international migrants from sub-Saharan Africa who are living with HIV. We address the question: “How do legal, policy, and socio-structural factors influence access to HIV care for African migrants, and what rights-based strategies can ensure health equity for this population?” We synthesize peer-reviewed and grey literature (2005–early 2025) using the Availability, Accessibility, Acceptability, and Quality (AAAQ) human rights framework integrated with a structural violence (SV) lens. We find that African migrants living with HIV encounter considerable systemic barriers to care driven by restrictive immigration policies, fragmented and exclusionary health financing, intersecting stigmas (racism, xenophobia, HIV stigma, homophobia), and inconsistent provider competencies. We recommend seven bold, yet actionable, strategies to align policy and practice with states’ human rights obligations: decoupling healthcare from immigration enforcement, tapping migrant community power, creating centralized migrant-inclusive health information systems, re-imagining migrant-inclusive universal healthcare, shared financial responsibility for migrant health, operationalizing ethical safeguards to protect the healthcare rights of migrants and recognition of the needs of uniquely situated migrants, particularly women, youth, and LGBTQI+ individuals.

## Introduction

International migration has long been an integral part of human history, shaping economies and fostering economic growth worldwide. As journalist Lydia Polgreen aptly stated, “Our world is made by movement — and there’s no way to stop it” (1). In today’s globalized world, migration is deeply interconnected with trade and investment, reflecting the growing interdependence of nations (2). Migration continues to be a defining feature of the 21st century. By 2024, an estimated 304 million people — nearly 4% of the world’s population — were international migrants (3). Of these, 60% were labor migrants, and nearly half (135 million) were women (4). The population of African-born migrants living outside the continent more than doubled between 1990 and 2020, from about 7.5 million to 19.5 million, and most African-born migrants now reside in Europe (11 million), Asia (5 million), and North America (3 million). Even so, movement *within* Africa remains substantial. United Nations (UN) estimates show an increase from 12 million in 2015 to 15 million in 2024 in the number of African migrants (excluding refugees and asylum seekers) living in other African countries (4–6). This migration is primarily driven by economic and political instability, including the need to escape violence, conflict, human rights violations, climate change, and war (7, 8).

Migration status is increasingly being recognized as a social determinant of health, with migrants often facing a disproportionately higher risk of ill health than host populations. Special calls in leading journals have urged the adoption of rights-based approaches to ensure that all individuals, regardless of migration status, can access health services (9–11). This aligns with the Sustainable Development Goals, notably SDG 3.8 on universal health coverage, and the 2024 Joint UN Programme on HIV/AIDS (UNAIDS) theme, “Take the Rights Path” (12, 13). Yet profound contradictions persist between legal commitments and practice, with migrants facing anti-migrant rhetoric, xenophobia, and structural obstacles to accessing healthcare that undermine their right to health (14). In the current global migration landscape, many migrants endure perilous journeys and arrive in destination countries in severe psychological and physical distress and in dire need of immediate and long-term healthcare (15). Simultaneously, globalization has contributed to the emergence and re-emergence of infectious diseases, highlighting the urgent need for effective public health responses for both migrants and host communities.

Some of the most vulnerable migrants are those living with HIV, yet little is known about their access to HIV care in host countries. Currently, there is no comprehensive, centralized global source on migrant-related HIV data (16). Furthermore, mobility-driven HIV transmission is common in many sub-Saharan African (SSA) countries. While such evidence highlights the scale of the problem, it provides limited insight into the structural and social dynamics that underpin migrant vulnerability to HIV infection.

Understanding the historical trajectory of the global HIV response is essential to contextualize the persistent inequities faced by African migrants living with HIV. From the outset of the epidemic, migrants have been entangled with HIV in ways shaped as much by politics as by health. For example, in 1987, the U.S. denied visas and legal status to people living with HIV and only lifted this ban in January 2010 (17, 18). Furthermore, HIV in the U.S. was initially associated with immigrants from Haiti, thereby racializing the disease from the start (18). Similarly, in Africa, HIV was initially associated with highly mobile populations and labor migration, with miners, truck drivers, and sex workers stereotyped as key drivers of the disease (136, 137). Several countries still place various restrictions on immigration for people living with HIV: 12 countries deport non-nationals on the basis of HIV, 11 countries may deny short and long-term stays on the basis of HIV, and 17 countries require HIV testing for certain types of permits (19).

An ever-growing body of research continues to document migrants’ heightened HIV vulnerabilities and unmet needs for HIV care. This narrative review examines healthcare access among migrants from SSA (hereafter referred to simply as “African migrants”) living with HIV. SSA accounts for two-thirds of the global HIV burden, with an estimated 26–36 million people living with HIV in 2024 (20, 21). Against this backdrop, we ask how legal, policy, and socio-structural factors influence African migrants’ access to HIV care and what rights-based strategies can be leveraged to advance health equity. We argue that a rights-based approach must be explicitly political, holding states and institutions accountable for structural barriers. Drawing on a structural violence lens, we interrogate how racism, sexism, economic systems, and colonial legacies affect access to HIV care.

## Theoretical framework: human rights and structural violence

Several foundational documents outline the path to realizing the right to health and other human rights. The International Covenant on Economic, Social, and Cultural Rights (ICESCR) (adopted by the UN Assembly in 1966 and enforced since 1976) provides a broad framework for key rights that states are required to recognize and exercise (22). These include rights to fair working conditions, social security, adequate living standards, education, healthcare, and cultural participation. The ICESCR recognizes the right to the highest attainable standard of health, which was subsequently elaborated in General Comment No. 14. It outlines states’ obligations to ensure the availability, accessibility, acceptability, and quality of health services (AAAQ) without discrimination, including prioritizing the needs of vulnerable populations and marginalized groups. The ICESCR acknowledges that the human right to health extends beyond healthcare to include underlying social determinants of health, such as access to safe water, adequate food, housing, and a healthy environment (23).

However, the implementation of ICESCR relies largely on state monitoring and international cooperation. Despite international frameworks that affirm the right to health for all, the realization of these rights, especially for people living with HIV, is frequently undermined by restrictive immigration policies, stigma, and the lack of policy coherence across borders. This challenge was articulated more than 20 years ago by UNAIDS in its 2001 seminal report, “Migrants’ Right to Health”, which highlighted gaps in coverage and accessibility and inadequate legal and policy frameworks. It also recognized the disconnect between international human rights commitments and their implementation in practice. Access to HIV care remains a critical challenge for migrants who often face structural, legal, and social barriers that compromise their health and human rights.

**Figure 1:**
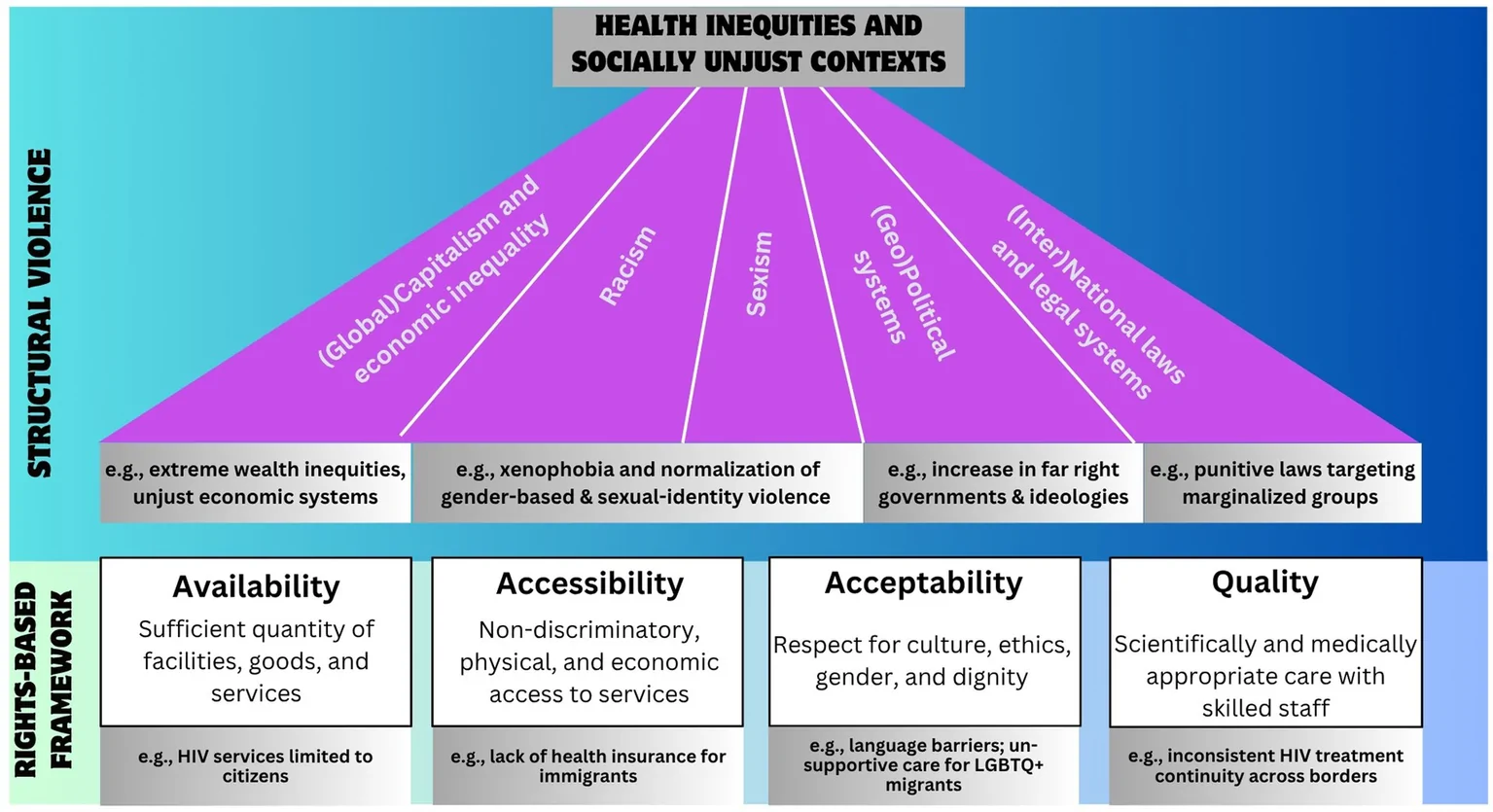
The AAAQ + SV health rights framework.

We applied the AAAQ human rights framework and integrated a structural violence perspective (AAAQ + SV) in this narrative review. We hope to show how unjust social structures constrain each AAAQ dimension, thereby contributing to and compounding health inequities. Anthropologist and medical doctor, Paul Farmer, argued that social inequalities are at the heart of structural violence and called for a historically grounded analysis of how “larger social forces”, such as racism, sexism, economic exploitation, political systems and colonial legacies, contribute to illness and disease (24) (see Figure 1 for a mapping of constructs onto the AAAQ). Structural violence is especially pernicious because unjust social structures are often normalized and treated as inevitable, so they go unquestioned. While there are similarities between the concept of structural violence and the social determinants of health (SDoH), we concur with several scholars who argue that the SDoH framework has lost its initial focus on social justice and has become increasingly sanitized and apolitical (25, 26). Medical anthropologist Yates-Doerr (2020, p.380), in particular, argues that the language of “determinants” prioritizes the identification of measurable, predictable contributors to ill-health and does not go far enough to advocate for deep structural transformation (25). For Hasemann Lara et al. (26), the SDoH framework tends to “proffer neoliberal solutions that obscure much of the violence and dispossession that influence contemporary migration and health-disease experiences” (26). Our AAAQ + SV framework encourages readers the reader to *also* consider the underlying “social machinery of oppression” (24) and the human decisions that facilitate the violation of human rights and sustain health inequities.

As Figure 1 shows, to effectively address health inequities as human rights violations, we will need to grapple with difficult issues, such as economic structures that privilege profits over people and enduring legacies of colonization that continue to shape the distribution of resources globally and foment racism and sexism. The experiences of African migrants in host countries should be understood within this broader geopolitical and historical context, rather than solely through a narrow focus on individual-level factors.

## Methodology

We conducted a hermeneutic narrative review since our goal was to generate deeper conceptual insights and actionable recommendations for improving HIV care for African migrants, rather than to catalog and count all available studies on African migrants and HIV care. Hermeneutic narrative reviews emphasize iterative cycles of searching, reading, and interpreting, allowing for a more flexible and critical engagement with diverse sources, especially for complex issues such as migrant health and well-being. Our evidence-informed review thus not only synthesizes available data but also offers new ways of thinking about how policies and strategies can be more responsive to migrants’ needs. Narrative reviews are particularly useful to both practitioners and policymakers, as they provide new insights or ways of thinking about a topic, integrate perspectives across different fields, and distill lessons that can directly inform practice and decision-making (27). Our synthesis highlights recurring themes, persistent knowledge gaps, and directions for policy innovation (28, 29).

### Search strategy and data sources

Our initial search used the terms “African migrants and HIV” and “African migrants and HIV care” across multiple databases, including PubMed, Scopus, Wiley, and Google Scholar, and variants of “sub-Saharan Africa” to identify relevant empirical publications.

We then iteratively expanded our search strategy to include 13 targeted Elicit searches, an AI-based research tool. Furthermore, we screened reference lists of published articles and reviewed grey literature, namely reports and policy documents from organizations such as the International Organization for Migration (IOM), the International Labour Organization (ILO), Human Rights Watch, Amnesty International, and UNAIDS. As new themes emerged, we conducted more focused searches on topics such as African LGBTQI+ migrants living with HIV, barriers to sexual health communication, xenophobia, HIV care policies for international migrants, ethical and implementation-science issues in migrant research, and human rights and health equity frameworks to explore these issues in greater detail. Importantly, identifying gaps in theoretical frameworks prompted targeted searches that strengthened the conceptual foundation of our analysis.

While our initial keyword search yielded 334 results, searches across additional platforms generated thousands more. Because this is a narrative review, our aim was not to capture all published articles but to identify literature that best illuminated gaps in HIV care and actionable insights to strengthen programs and policies for African migrants living with HIV. Using an iterative, evidence-informed process guided by theoretical and thematic saturation (30), we identified 137 articles that meaningfully informed our analysis.

To facilitate robust collaboration, we set up a shared Dropbox folder accessible to all team members and stored all potentially relevant articles there. Between March and September 2025, our five-person team met bi-weekly to review emerging themes, compare findings, and determine whether additional searches were needed. We concluded that thematic saturation had been reached when team members independently reported similar themes, and no new conceptual insights were emerging from the literature.

### Scope of the review

We included quantitative, qualitative, and mixed-methods studies published in English from 2005 onward, aligning with the World Health Organization’s (WHO) “3 by 5” initiative — which aimed to ensure that 3 million people living with HIV were on HIV treatment by 2005 — marking the global scale-up of antiretroviral therapy in Africa. For this review, we defined international migrants as individuals who cross national borders to live or work in another country (31). We excluded studies that focused on non-African migrants, internal migrants, refugees, populations experiencing external forced displacement, or children. We focused specifically on migrants from SSA as the subregion accounts for most HIV infections globally.

### Multidisciplinary research team

Our multidisciplinary author team spans public health, global health, medical anthropology, health economics, epidemiology, and bioethics. Most of the authors have been working in HIV programming and research for at least two decades, including with individuals from SSA and African migrants, specifically. This positionality informed our study selection, interpretation, and rights-based analytic stance.

## Analysis

### Key timelines in global HIV care trajectory

**Figure 2:**
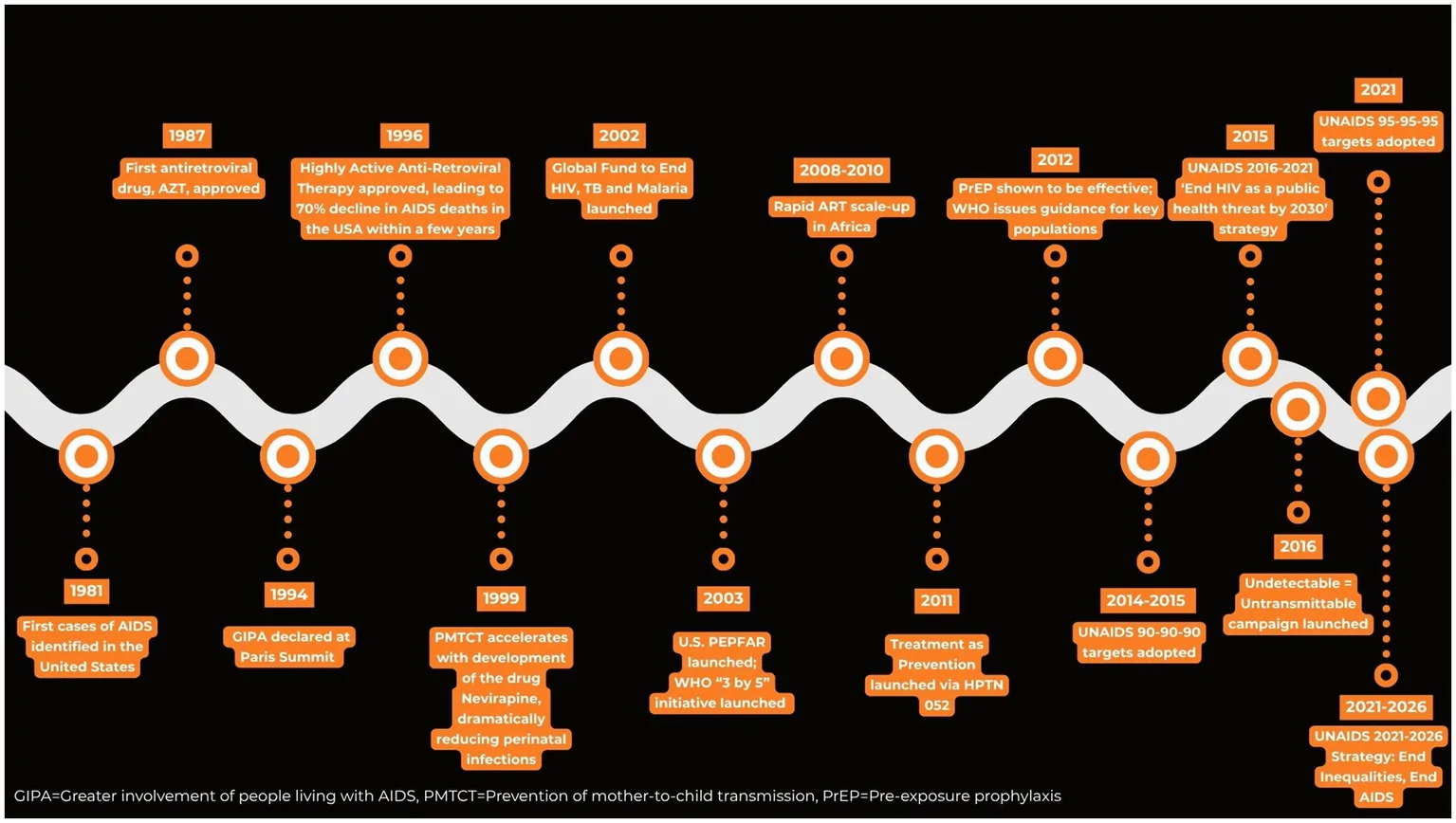
Timeline of key events in the HIV trajectory.

We begin this narrative review with Figure 2, which presents a timeline of key events in the HIV trajectory. The spacing between events is not intended to reflect the time elapsed between them. These events have shaped current access to HIV treatment and care among African migrants living with HIV and situate their current realities within broader global and regional shifts. While this review focuses on the period between 2005 and 2025, earlier milestones are included to illustrate how foundational breakthroughs in HIV science, activism, and financing shaped subsequent access to treatment and care for African migrants living with HIV. Highlighted developments include the “3 by 5” initiative, which was a global compact led by the WHO to ensure that 3 million people living with HIV from low- and middle-income countries would be on treatment by the year 2005; this initiative was made possible through the U.S. President’s Emergency Plan for AIDS Relief (PEPFAR), which devoted hundreds of millions of dollars towards the procurement of HIV medications (32). Since then, major scientific, political, and programmatic milestones have altered HIV treatment and care worldwide, transforming HIV into a treatable condition. However, these advances have not been equitably distributed, and structural barriers, such as restrictive migration policies, limited health systems, and stigma, have continued to shape migrants’ experiences. It is important to recognize that HIV has been a treatable and manageable chronic condition for more than two decades, with viral suppression shown to *prevent* transmission to uninfected partners during condomless sex (33, 34). This remarkable effectiveness in contemporary HIV medications, coupled with the widespread availability of HIV medications due to PEPFAR funding, makes the denial of HIV care to migrants in host countries especially disheartening.

### Intersecting vulnerabilities and barriers to HIV care for African migrants

A large body of evidence demonstrates that African migrants experience a disproportionate burden of HIV both before and after migration, shaped by social, economic, and structural inequalities. Multiple reviews show markedly higher HIV prevalence among migrants from SSA compared to migrants from other regions, with prevalence ranging from 3.7 to 3.9% among migrants from Southern, Central, and East Africa, and far lower rates (typically under 1%) among migrants from Eastern Europe, Latin America, and Southeast Asia (35, 36). These disparities persist across host countries. SSA migrants accounted for 37% of new HIV diagnoses in France in 2018, 58% of new infections in Portugal in 2017, and had more than three times the odds of HIV infection in Spain (16). Across the European Union and European Economic Area, nearly half of all new HIV diagnoses in 2023 were among migrants, with migrants from SSA experiencing a 20.4% increase over the previous year (37).

A recurring question in the literature is whether African migrants acquire HIV before or after migration. Studies suggest that both pathways are common. Harsh socio-economic conditions, unstable housing, and exclusion in host settings have been strongly associated with post-migration HIV acquisition, as seen among SSA migrants in France (38). Conversely, other studies in the U.S. show that most infections among some African migrant populations were acquired prior to migration (e.g., 69% pre-migration compared to 29% post-migration in a sample of 179 African migrants) (39). Migration itself can also be shaped by HIV. This was the case in the 1990’s and early 2000’s among individuals who migrated from Africa to the Global North in hopes of accessing HIV medications which were either unavailable or too expensive in African countries (40). Following the success of the Treatment Action Campaign in lobbying for cheaper generic HIV drugs in South Africa in the early 2000’s, migration to South Africa and Botswana increased as individuals tried to access these otherwise elusive medications (41).

Poverty, labor market exclusion, and restrictive immigration regimes further heighten migrants’ vulnerability to post-migration HIV infection (42). Many migrants, regardless of documentation status, struggle to secure employment upon arrival, encounter work restrictions or prolonged permit-processing delays, and often end up in low-wage, physically demanding, or informal-sector jobs. Many countries impose work restrictions on migrants or take too long to process work permits, leaving many unemployed or precariously employed in jobs that entail hard physical labor, long work hours, and minimum or below minimum wage (4, 43, 44). Under these circumstances, some resort to sexual–economic relationships, including exchanging sex for housing or food (38, 45). Studies in Southern Africa have long shown that mobility, labor exploitation, and constrained livelihood options intersect to shape exposure to HIV within broader structural conditions (46). These realities underscore that migrants’ vulnerability is not simply a matter of individual behavior, but a predictable outcome of structural violence, embedded in immigration policy, labor markets, and social exclusion (47, 48).

The ethical issues surrounding migration pose a challenge to health equity for international migrants (49). Public health ethics provide a complementary normative foundation for this analysis, reinforcing why migrant access to HIV care is not only a legal obligation but an ethical imperative (50). Core principles of public health ethics include justice, equity, solidarity, reciprocity, and the duty to prevent avoidable harm and are particularly salient in the context of migration (51). Justice and equity require that health systems prioritize populations whose vulnerability is structurally produced through legal exclusion, precarious legal status, and social marginalization (50). Solidarity recognizes that health risks and benefits are shared across populations and borders, while reciprocity underscores the ethical obligation to protect migrants who contribute socially and economically to host societies yet are often denied full access to care (52). From a harm-prevention perspective, restricting or delaying HIV diagnosis and treatment for migrants is ethically indefensible, as it predictably results in preventable morbidity, mortality, and onward transmission, outcomes that violate foundational public health obligations to minimize avoidable harm (50, 51). Together, these ethical commitments highlight the moral limits of sovereignty in public health decision-making and provide a normative rationale for evaluating whether health systems meet their obligations to migrant populations (52).

Within this context of heightened vulnerability, the barriers African migrants face in accessing HIV care take on deeper significance. The concept of structural violence is critical here. Exclusion from labor protections, lack of documentation, and limited political voice are not accidental but institutionalized conditions that entrench vulnerability (53). Applying the AAAQ + Structural Violence (SV) framework helps illustrate how states’ obligations to ensure the availability, accessibility, acceptability, and quality of HIV care are routinely undermined by systemic failures. We begin by discussing each element of the AAAQ and conclude by highlighting the implications for structural violence.

#### Availability: unequal provision of HIV services for migrants

Availability in the AAAQ framework refers to the type and quantity of facilities, goods, and services. Availability remains uneven because HIV services for migrants are often fragmented and contingent on immigration status, geography, or local policy. In many countries, restrictive immigration policies exclude migrants from public health programs or limit them to emergency care. In Europe, only 21 of 48 countries provide some level of HIV care to undocumented migrants, and where such services exist, they vary greatly in scope and sustainability, and continuity is complicated by migrant mobility (54). Frequent cross-border travel in Southern Africa, for example, routinely interrupts antiretroviral therapy (ART), and weak regional coordination prevents consistent access to medication. In Mozambique, frequent travel among migrants living with HIV disrupted continuity of ART for up to 2 months (55). Similar patterns have been reported in West Africa, where mobile populations crossing porous borders faced service disruptions due to the absence of harmonized regional HIV protocols. Collectively, such gaps represent clear departures from international human rights guaranteeing continuous and equitable HIV service provision for migrant populations.

#### Accessibility: legal and structural barriers to HIV care

Within the AAAQ framework, accessibility encompasses physical accessibility, financial affordability, non-discrimination, and access to the information needed for equitable healthcare. In practice, this means that health services must be within safe physical reach, affordable, and available to all without discrimination. Yet, for African migrants living with HIV, accessibility poses a challenge. Although international obligations affirm that healthcare should not depend on citizenship or immigration status, as reflected in Article 12 of the ICESCR and General Comment 14 (23), in practice, many countries, both within and outside Africa, provide comprehensive HIV services only to legal or documented migrants, typically through government-run facilities. In Denmark and Australia, eligibility varies by residency or visa category (56), leaving groups such as international students uncertain about entitlements and hidden costs (57).

Many health systems embed “everyday bordering” practices, i.e., ongoing strategies through which states exercise power and regulate diversity, often resulting in discriminatory treatment of migrants (58). Lim distinguishes between two forms of discrimination: *policy-based discrimination,* stemming from explicit policy decisions targeting specific migrant groups, and *procedure-based discrimination*, arising from the barriers migrants encounter while navigating step-by-step migration or service processes (59). Within the context of healthcare, such discrimination is enacted by requiring proof of legal residence for registration, linking health records to immigration databases, or restricting care to emergency services (60, 61). These measures deter migrants from seeking diagnosis, worsen treatment outcomes, and undermine public health goals.

South Africa offers a stark example of the persistent gap between legal entitlements and lived realities. Although its Constitution (Section 27) enshrines the right to health for all (2), undocumented migrants, as in the case of those from Zimbabwe, are frequently denied services or charged unaffordable fees (62) under the principle of “progressive realization” which allows the state to limit services based on resource constraints. Fear of arrest or deportation deters many migrants from seeking care, especially when healthcare access is linked to immigration enforcement (60, 63, 64). Healthcare providers often lack clarity about migrants’ entitlements and fear violating regulations tied to documentation requirements, and believe they are legally unable to treat patients without proper identification, even in cases of severe illness. These gaps have real health consequences.

**Figure 3:**
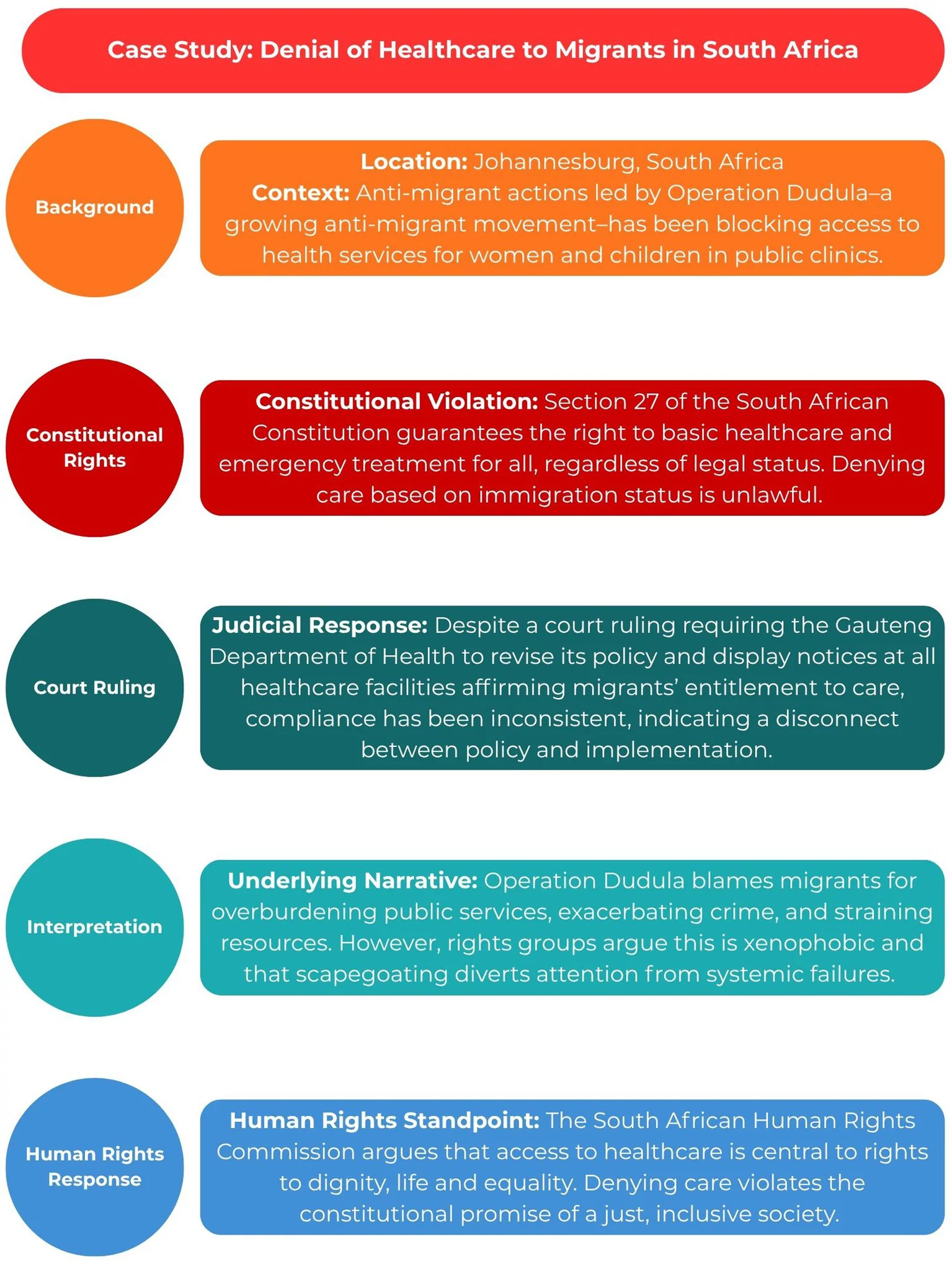
Case study. Denial of healthcare to migrants in South Africa.

Operation Dudula, a growing anti-migrant movement in South Africa, has been actively obstructing migrants’ access to healthcare services in public clinics (see Figure 3), sometimes harassing pregnant women, forcing them to stand in separate queues from South African nationals, and blocking their entry to clinics. In response to these discriminatory actions, a court ordered the Gauteng Department of Health to revise its policies and ensure compliance with constitutional protections. The ruling specifically required that notices be displayed in all healthcare facilities, affirming that lactating women and children may not be denied care. Despite this legal intervention, implementation has been inconsistent, highlighting a persistent gap between policy and practice, further entrenching a climate of fear, xenophobia, hate speech, unlawful vigilantism, and uncertainty. Operation Dudula justifies its actions by claiming that undocumented migrants overburden the public healthcare systems, fuel crime, and contribute to unemployment and poverty. Such rhetoric scapegoats migrants and diverts attention from the government’s failure to address deeper structural weaknesses within the health and social systems. The South African Human Rights Commission has urged the Department of Home Affairs and the South African Police Service to fulfill their responsibilities to prevent citizens from taking the law into their own hands. The Commission further stresses that access to healthcare is not only a legal right but a moral imperative central to the rights to dignity, life, and equality (65).

Even when HIV care is offered on humanitarian grounds, gaps in implementation remain (66). Across Europe, access to HIV services is often tied to visa status, leaving asylum seekers, international students, and migrant workers vulnerable to gaps in care. Several non-EU countries offer free healthcare to undocumented individuals through their national health insurance programs. Since 2015, Israel has been providing free HIV care to undocumented African migrants through its national health system, yet its implementation remains uneven (67). Undocumented African migrants in New York City (NYC), for example, often delay initiating HIV care due to fear of immigration enforcement, difficulties obtaining health insurance, beliefs about ineligibility for care, social isolation, and HIV-related stigma (68). Another NYC study echoed these findings, identifying fear of deportation and stigma tied to undocumented status as key barriers to both initiating and staying in care (69).

For most undocumented migrants, however, the challenges are even greater. Many are excluded from public health insurance schemes or must pay out-of-pocket costs far beyond their means, pushing them to rely on non-governmental organizations or informal networks. In South Africa, undocumented Malawian migrants sometimes rely on contacts in Malawi to collect and send ARVs across the border, purchase drugs from informal “street pharmacies”, or depend on friends with connections in the health system (70). Media reports also highlight cases of Malawian migrants turning to drug smuggling syndicates to obtain ARVs at exorbitant prices to avoid treatment interruptions. Multi-month dispensing of ARVs has improved continuity in some African countries. For example, in Lesotho, a survey of 524 migrants in Lesotho’s border districts found that 93% preferred to collect their HIV medication in Lesotho, with most requesting at least three-month refills to cover their time in South Africa (71). Multi-month dispensing of HIV treatment in Lesotho is generally restricted to 6 months. Limited opportunities for migrants working in South Africa’s informal economy to travel back to their home countries mean that treatment interruptions are common, leaving migrants at risk of advanced HIV disease (72).

Thailand stands out as a rare example where free universal healthcare, including HIV services, are legally extended to all migrants regardless of documentation status (73). Programs such as the Migrant Health Insurance Card Scheme and the One-Stop Service Registration aim to extend coverage to undocumented workers. However, implementation gaps persist (74–76). In Ranong province, qualitative interviews with policymakers and healthcare providers identified several systemic challenges. Slow, inefficient nationality verification processes, poor communication of policies, bureaucratic delays, clinic hours that conflict with migrants’ work schedules, poor inter-ministerial coordination, and fear of arrest often prevent migrants from enrolling or consistently using available services.

Several studies note that documentation status is often fluid and precarious. Migrants may lose legal residency due to expired visas, delayed processing, or rejected asylum claims, which can abruptly cut off access to healthcare. A study of sexual and gender minority migrants in South Africa found that individuals lost access to healthcare when their asylum applications were rejected, even while appeals were pending (77). Similar patterns have been documented among African migrants in the United Kingdom (78). Ultimately, these legal and structural barriers violate the principles of equity and non-discrimination in healthcare and undermine both global and national commitments to HIV care. Addressing these gaps will require immediate policy coordination and long-term reforms to harmonize health and immigration systems and ensure that access to HIV care is truly universal.

#### Acceptability: cultural, linguistic, and stigma-related barriers

Acceptability requires that health services respect cultural differences and medical ethics and be responsive to patient needs. Many African migrants encounter culturally insensitive or linguistically inaccessible care environments, a lack of professional interpretation services, judgmental provider attitudes, and inadequate psychosocial support, which erode trust and discourage disclosure of sexual health concerns. Language barriers are common, and interpretation services — when available — are often informal, leading to breaches of confidentiality and reduced care quality. Studies show that African migrants in European countries frequently report discomfort in discussing sexual health issues due to judgmental provider attitudes or culturally inappropriate counselling (79). Additionally, a study of African migrants living with HIV in the U.S. found that they face multiple intersecting stigmas due to being Black *and* African *and* multilingual (80). The authors note that these characteristics represent historically socially oppressed identities in the U.S. and negatively affect African migrants’ interactions with healthcare systems and providers.

Nöstlinger et al. (81) identified inadequate attention to cultural norms and the absence of psychosocial support as deterrents to care-seeking. These challenges are intensified for migrants identifying as lesbian, gay, bisexual, transgender, queer, or questioning, who face triple stigmatization based on HIV-positive status, sexual orientation and immigration status. Consequently, many avoid clinics entirely due to previous experiences of homophobic or medical xenophobic treatment (82, 83). This discrimination may appear in language, treatment, or even the denial of healthcare services. Survivors of sexual violence, often common among female migrants and gender minorities, may find themselves retraumatized in uncaring or poorly informed service environments and thus defer engagement with care (84). Such experiences violate respectful, dignified healthcare and reduce prospects for early diagnosis or sustained treatment.

#### Quality: gaps in provider training and health system responsiveness

The principle of quality demands that health services are scientifically appropriate and delivered by trained professionals in systems that are responsive to patients’ needs. Yet, quality gaps persist due to limited provider training on migration-related vulnerabilities, fragmented health information systems, and overstretched health clinics that deprioritize migrant-inclusive services (85). Provider ignorance of migration-specific stressors, such as trauma, disruption of ART regimens, and social isolation, results in care that is neither tailored nor effective (86), which leads to long wait times, minimal counselling, and inconsistent ART provision. Studies show that mobile populations often become virally unsuppressed because of treatment interruptions, particularly in settings where national HIV databases cannot be linked across borders (87). Few countries have clinical protocols that address the intersecting vulnerabilities of migrants, for example, mental health support for those living with HIV or integrated reproductive health services for undocumented women. Without migrant-specific competencies, health systems fall short of delivering the high-quality care required under human rights obligations.

In sum, these intersecting factors show that African migrants’ heightened vulnerability to HIV and persistent barriers to care are intertwined. The structural factors that increase HIV risk – poverty, precarity, discrimination, and exclusion – are the same forces that limit access to treatment and adherence. A human rights-based approach demands addressing not only the gaps in availability, accessibility, acceptability, and quality of services, but also the broader systems of structural violence that constrain migrants’ lives and health trajectories.

## Structural violence and intersecting discrimination

A structural violence lens helps explain how social systems, policies, and institutions create, reinforce, and sustain inequities. Many of the barriers to HIV care for African migrants are intensified by underlying systems of inequity tied to racism, sexism, harmful economic systems, and neocolonial geopolitical realities. A review of studies conducted among U.S.-based Black African adult immigrants from five countries found that having experienced racism was consistently associated with poorer mental, behavioral, and physical health outcomes, which mirrored the health experiences of non-migrant African Americans who encounter racism in many aspects of American society due to the enduring legacies of slavery and 1960’s Jim Crow racial segregation laws (88). Another study shows growing numbers (72%) of immigrants in the U.S. who identified as Black in 2018; as this population becomes more visible, their vulnerability to racial discrimination also increases (88, 89). Many anti-immigrant sentiments reveal deep-rooted fears of demographic shifts in the racial, ethnic, and cultural composition of countries in the Global North, which are predicted to have majority non-white populations in the foreseeable future (90). These concerns are central to the growing popularity of far-right ideologies, and are shored up by deep-rooted beliefs in the superiority of whiteness as a social, cultural, economic, and political phenomenon (91). Such beliefs — and the institutions that legitimize them — make it easy to dehumanize migrants and deny them access to basic human needs such as health and HIV care. The intersection of race and immigration status intensifies structural disadvantage, fueling stigma and leading to sub-optimal health outcomes.

Similarly, when viewed from a structural violence lens, xenophobia is ultimately about ethnocentrism, that is, the belief in the superiority of one’s own ethnic identity (92). Studies show that xenophobia is not directed at all foreigners equally but often targets specific groups perceived as ethnically inferior in some way. Xenophobia, therefore, is fundamentally about (re)asserting group dominance over others and is often enabled and reinforced by social institutions and policies that ignore or tacitly encourage anti-immigrant sentiment by not penalizing citizens who commit acts of violence against migrants. African migrants typically constitute racial, ethnic, and religious minorities in host countries and are thus marginalized, which underscores the need to address xenophobia as a human rights issue — not merely as an economic concern nor an isolated activity undertaken by criminals. Xenophobia reflects systemic power imbalances — not merely individual beliefs — that privilege certain linguistic and racial/ethnic groups over others, resulting in the extreme surveillance of, and violence toward, migrant populations (92). Relatedly, heteronormativity as a social system makes it easy to deny gender and sexual minorities easy access to healthcare services, and this is magnified when these groups are also racial or ethnic minorities.

## The way forward: bold, actionable strategies

**Figure 4:**
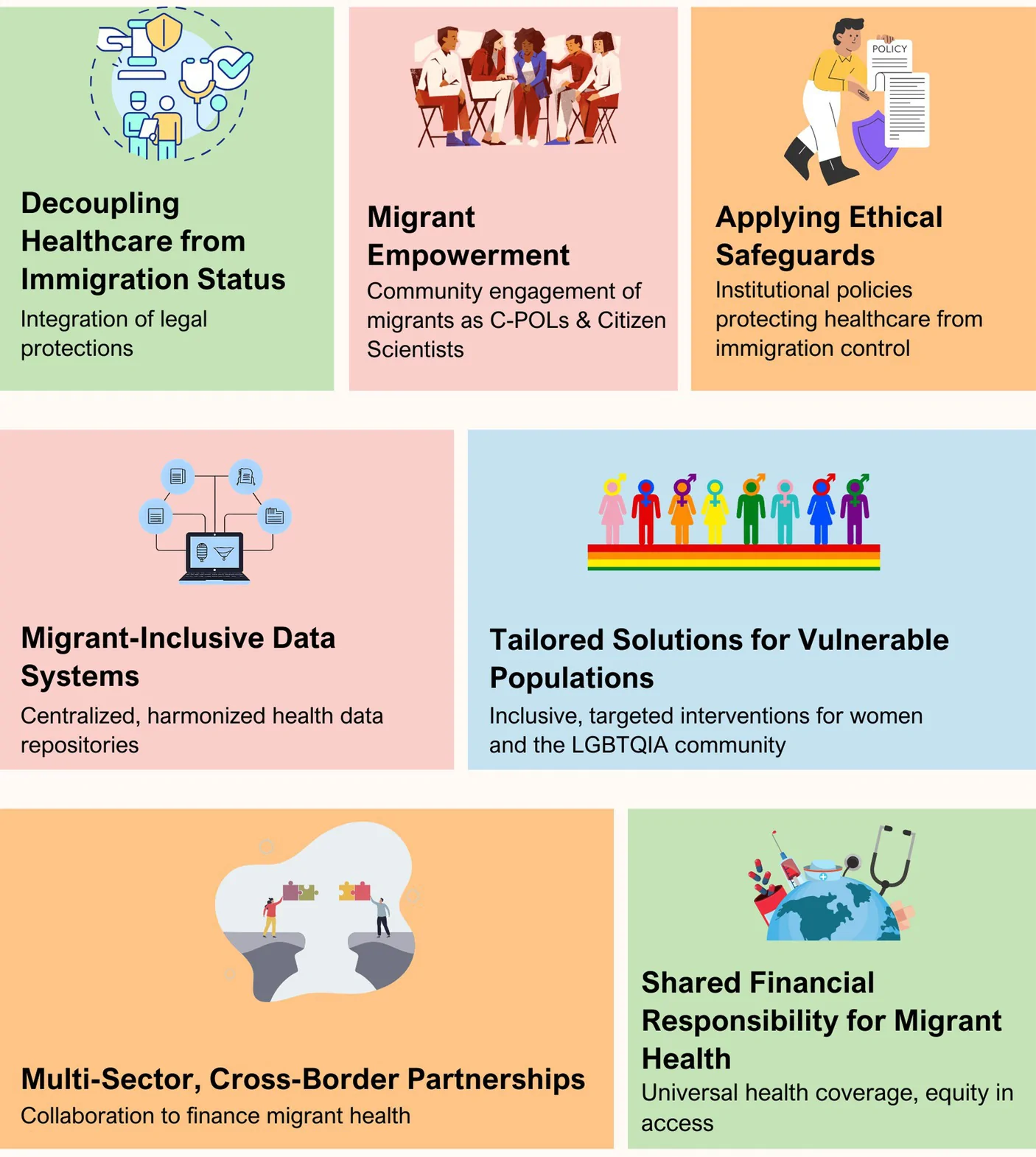
Bold, actionable strategies.

Below we offer seven bold, yet actionable, strategies for protecting the healthcare rights of African migrants living with HIV (Table 1 and Figure 4). Each of these strategies is a response to systemic failures in addressing the rights embedded in the AAAQ framework and in tackling structural violence to ensure that health is indeed a human right for all, including migrants. The strategies are grounded not only in international human rights law but also in widely accepted principles of public health ethics, including justice, solidarity, reciprocity, and harm prevention, which together demand collective responsibility for safeguarding the health of migrants.

**1 Decoupling healthcare from immigration status and integrating legal protections into health policy**

**Table 1:** Summary of bold, actionable strategies by AAAQ + SV health rights framework.

Suggested strategies	Summary	AAAQ + SV components addressed by strategy
Decoupling healthcare from immigration status and integrating legal protections into health policy	Shifting political attitudes to acknowledge economic and political drivers of migration	Strategy addresses *accessibility* (removing immigration-linked barriers) and *acceptability* (ensuring non-discriminatory treatment) barriers, while directly confronting structural violence in laws that criminalize or exclude migrants.
Tapping community power and meaningfully engaging migrants	Recognizing migrants as agents for promoting health equity	Strategy addresses *acceptability* (culturally congruent, stigma-reducing approaches) barriers and counters structural violence by redistributing power and knowledge production from elite institutions to migrant communities.
Operationalizing ethical safeguards to protect healthcare rights of migrants living with HIV	Creating institutional policies that protect healthcare provision from immigration control, promoting the privacy of patients	Strategy addresses *accessibility* (ensuring healthcare is safely accessible for migrants) and *acceptability* (enhanced provider training on cultural humility and anti-racism to promote institutional accountability).
Creating centralized, migrant‑inclusive digital health information systems	Creating a digital migrant health data system to promote continuity of care	Strategy addresses *availability* (continuous data on HIV services), *accessibility* (cross-border information continuity), and *quality* (improved provider responsiveness) barriers, while confronting structural violence by dismantling fragmented systems that exclude migrants.
Reimagining universal healthcare coverage in the context of migration	Utilizing rights-based, comprehensive healthcare programs that are widely accessible to migrants	Strategy directly targets *accessibility* (legal access to services) and *availability* (entitlement to essential HIV care) barriers. It counters structural violence by challenging exclusionary citizenship-based healthcare models.
A shared responsibility for financing healthcare for migrants	Fostering multi-sectorial governing structures that promote universal healthcare coverage	Strategy addresses *availability* (ensuring sustained ART, PrEP, and testing programs) and *quality* (avoiding treatment interruptions due to funding gaps) barriers. It confronts structural violence by challenging exclusionary citizenship-based financing models and shifting responsibility from resource-poor host countries to equitable global pooling mechanisms.
Developing solutions that cater to the specific needs of uniquely situated migrants like women, youth and lesbian, gay, bisexual, transgender, queer, or questioning (LGBTQI+) individuals	Progressing solutions that target the unique needs of various migrant populations who face overlapping inequities	Strategy strengthens *acceptability* (services tailored to cultural, gender, and identity needs) and *quality* (specialized provider competencies). It addresses structural violence at the intersections of sexism, heteronormativity, and racism.

This strategy addresses accessibility (removing immigration-linked barriers) and acceptability (ensuring non-discriminatory treatment) barriers, while directly confronting structural violence in laws that criminalize or exclude migrants.

Decoupling healthcare from immigration status requires more than administrative reform. It demands a shift in political attitudes and an acknowledgement of the enduring colonial legacies that shaped Africa’s borders and continue to drive inequities today. Host-country governments must also reckon with Africa’s geopolitical and economic vulnerabilities, which are compounded by the extractive capitalist actions of players based in the Global North, where most host countries are often located. A 2024 World Bank Report underscores this disparity: while extreme poverty has decreased globally since 1990, the number of people living on less than $2.15 per day in Africa has doubled (93). Furthermore, SSA now accounts for the world’s largest share of people simultaneously at high risk of extreme weather events and highly vulnerable to their impacts, even though 80% of global greenhouse emissions originate in wealthier countries. Within Africa, corruption further depletes scarce resources and deepens inequalities. Acknowledging that present-day migration is largely driven by unjust global and domestic social structures is an essential step toward humanizing migrants and holding politicians and corporations accountable.

Despite these geopolitical realities, migration policies in the Global North frequently prioritize surveillance and control over health rights. For example, the European Commission’s 2020 New Pact on Migration and Asylum aimed to balance responsibility-sharing with solidarity yet focused primarily on identification and fingerprinting, offering limited provisions for health screening (94). Four years later (April 2024), this Pact was made into law by the European Parliament and EU member states and included tighter screening procedures and carrying out health and security checks for entry into the EU, effective as of June 2026 (8, 84). The absence of an EU-wide health framework for migrants reflects a missed opportunity to align migration management with public health and human rights imperatives (81).

**2 Tapping community power and meaningfully engaging migrants**

This strategy addresses acceptability (culturally congruent, stigma-reducing approaches) barriers and counters structural violence by redistributing power and knowledge production from elite institutions to migrant communities.

Too often discussions about migrants living with HIV frame them as passive recipients of care or as problems to be solved. However, this view fails to recognize their agency and potential as catalysts of change. As cultural insiders, migrants could serve as powerful change agents with the potential to drive health equity when meaningfully engaged in the design and delivery of health services. One successful model is the Community Popular Opinion Leader (C-POL) strategy grounded in the diffusion of innovation theory (95). C-POLs are respected, socially influential individuals with strong community ties who can influence attitudes and behaviors of individuals in their social networks. Studies have shown that when C-POLs are activated, they can help improve both community and facility-based HIV services (96–98). Their effectiveness often stems from key network characteristics, such as their centrality, connectivity, the size of their social circles, and trust in opinion leaders, which can make them influential in disseminating new information and practices (99). However, rigorous evaluations of long-term impact and scalability in cross-border contexts remain limited. Moreover, there is a dearth of studies that have implemented C-POL interventions with African migrants living with HIV.

Another underutilized but innovative strategy is the use of Citizen Science models to engage migrants living with HIV to become non-professional researchers to observe, document, and analyze data (100–102). To build community research capacity, migrants living with HIV could partner with professionals and be trained to monitor migrants’ access to care and service quality and develop solutions grounded in the realities of migrant life. This would not only democratize data collection but also help to ensure that interventions are grounded in lived experience. Despite their promise, few studies have used citizen science methods to monitor access to HIV services among migrants. Yet the model holds considerable potential, especially in resource-limited settings, by empowering migrants to document service availability, barriers, and stigma, and using these data to guide program and policy design. A small study in Sierra Leone trained community health workers in ethnographic research and found that they successfully elicited local perspectives on vaccine confidence, supporting the feasibility of using community members with limited training to track information (103). Using citizen scientists to monitor and report on access to health services among African migrants living with HIV would be an innovative use of this potential community resource (104, 105).

In shifting our perspective, from viewing migrants as burdens to recognizing them as partners in change, we open the door to more sustainable, community-led solutions. These approaches value local knowledge, build on existing networks of trust, and, ultimately, create a more inclusive and effective public health response. Another example is the work of a community organization in the U.S., which has successfully used the “trusted messenger” model to collect information on the health equity concerns of community members in Central Massachusetts. The information is then used to develop a community health improvement plan that is overseen by the local department of public health (106)^.^ This integration into the local health infrastructure ensures that collected data are used to inform actual programs. Trusted messengers are respected individuals in their communities who are trained to collect health equity data via in-depth interviews, focus groups, and “community conversations” (106). Studies show that trusted messengers are more successful at getting community members to open up more during data collection than professional researchers, largely because they are from the communities being studied and often share similar experiences and look like the people they are collecting data from, in terms of race, class, gender, and *migration* status. We found relatively few examples of these participatory approaches with migrants. Arias Cubas and Mudaliar (107) critique the current state of international migration research, noting that it is primarily dominated by scholars based in high-income countries in North America and Western Europe. They advocate for greater inclusion of researchers with lived migration experience as well as those working directly with migrant communities in the Global South, including migrants themselves.

The exclusion of migrants from the design, implementation, and dissemination of research not only affects relationships of trust, collaboration, and reciprocity among migrants, researchers, and practitioners but also limits our collective understanding of their needs. Community-led and migrant-led interventions further illustrate the potential of rights-based approaches to transform HIV service delivery. Non-governmental organizations can advocate for reform of migration policies that restrict migrants’ access to healthcare services (19). Peer-led adherence clubs, such as those adapted from the C-POL model, harness trusted social networks to promote testing, linkage to care, and retention in care (108). However, we acknowledge that empowering migrants as citizen scientists, trusted messengers, or change agents must be accompanied by safeguards around confidentiality, stigma, and informed consent, particularly for undocumented populations.

**3 Operationalizing ethical safeguards to protect healthcare rights of migrants living with HIV**

This strategy addresses accessibility (ensuring healthcare is safely accessible for migrants) and acceptability (enhanced provider training on cultural humility and anti-racism to promote institutional accountability). This tackles structural violence by ensuring institutional-level change.

Building on our ethical and structural violence framework, a critical strategy for improving HIV care for African migrants is the operationalization of ethical safeguards within healthcare institutions and programs. Although legal and ethical norms affirm migrants’ rights to health, these commitments often fail to translate into routine practice, where legal precarity, language barriers, and concerns about immigration enforcement continue to affect access to care and accelerate disengagement from care. Closing this implementation gap requires clear institutional policies that separate healthcare provision from immigration control and protect the confidentiality of migrants’ health information, coupled with explicit communication of these protections to migrants (109). Institutional accountability should be strengthened by training providers in cultural humility, anti-racism, and migrants’ rights to address discrimination and power asymmetries in clinical encounters (110). In parallel, migrants must have access to safe, culturally sensitive, and multilingual mechanisms to report discrimination or mistreatment, with assurances of anonymity and protection from immigration scrutiny. Finally, embedding rights-based and ethics-centered approaches within HIV programs is not only a moral imperative but also a practical strategy to build trust, facilitate initiation of treatment, and sustain long-term engagement in care among migrant populations.

**4 Creating centralized, migrant-inclusive digital health information systems**

This strategy addresses availability (continuous data on HIV services), accessibility (cross-border information continuity), and quality (improved provider responsiveness) barriers, while countering structural violence by dismantling fragmented systems that exclude migrants.

A study of newly arrived migrants and refugees (within the first year of arrival) across 33 EU/EEA countries found that, since 2015, there has been little to no systematic data collection on their health status in the EU/EEA, making comparisons across countries extremely difficult. This gap was recognized over a decade ago in Rechel et al.’s (2012) review of migrant health data collection in Europe, and more recently by UNAIDS in its call for systematic monitoring of HIV and migration (111, 112). Given growing interconnectedness and the persistent lack of migrant-specific data, the development of multi-level health data information management systems at national, regional, and global levels is urgently needed.

We recognize that a single centralized global or regional migrant health database would be politically, legally, and ethically difficult to establish and maintain. Sharing non-anonymous, non-disaggregated health data across borders risks violating privacy laws, such as the General Data Protection Regulation (GDPR) in Europe, the Health Insurance Portability and Accountability Act (HIPAA) in the U.S., and the Protection of Personal Information Act (POPIA) in South Africa (113). Migrants may fear that such centralized health information systems could be used for immigration control rather than for health, raising questions about consent, data ownership, and whether migrants themselves have meaningful control over how their health records are shared. Trust in health systems is already fragile, and systems perceived as punitive could discourage care-seeking.

Moreover, migrants often move across multiple jurisdictions with diverse digital infrastructures and health data governance systems, creating additional legal and logistical challenges. One alternative is for migrants to carry their own portable digital health records (on a secure smartphone app, digital secure card, or blockchain-based “health passport”) that can be shared with providers at their destination with informed consent. A more feasible approach, however, may be interoperability: enabling national health information systems to “speak” to one another through standards such as the International Patient Summary and WHO’s Smart Guidelines (114–116). This would require bilateral or regional agreements to allow controlled data exchange when migrants cross borders. A federated model, in which countries query one another’s systems while keeping data at its source, would avoid the risks of centralized registries. To ensure trust, strict firewalls between health data and immigration enforcement would be essential, supported by rights–based agreements. Such systems would prioritize and normalize the consistent collection of disaggregated indicators to allow longitudinal tracking of service gaps and the effectiveness of migrant-focused interventions. Data could also highlight differences and similarities between migrants and host populations, and analyses of African migrant data should account for their racial, ethnic, and gender diversity, which shape HIV service access.

Existing initiatives provide useful models for such centralized systems. The EU Initiative on Health Security (2020–2025), for example, seeks to build regional capacity to address communicable disease threats and fosters cross-border cooperation, including with EU neighbors. While this could be extended to HIV among migrants, caution is warranted: public health and national security have at times been used in militarized ways that violated human rights, such as restrictions on entry for people living with HIV, AIDS denialism that delayed treatment access, and border closures during the 2014 Ebola outbreak and COVID-19 pandemic. It is for this reason that we intentionally refrain from using the word “surveillance” and instead prefer the more neutral term “health information system”.

Global initiatives such as PEPFAR demonstrate the value of standardized health information systems, although the future of its data strategies is uncertain due to termination of USAID. The European Centre for Disease Control and Prevention (ECDC) and its partnership with Africa CDC illustrate the benefits of harmonized disease monitoring and data sharing, thereby strengthening Africa CDC’s capacity for preparedness and response (117). Building on these models, a dedicated framework for migrant health information systems, integrating elements from PEPFAR, EU, and ECDC approaches, could enable comparability across countries, support cross-border collaboration, and promote equity-oriented responses to migrant healthcare needs, particularly for undocumented populations.

**5 Reimagining universal healthcare coverage in the context of migration**

This strategy directly targets accessibility (legal access to services) and availability (entitlement to essential HIV care) barriers. It addresses structural violence by challenging exclusionary citizenship-based healthcare models.

According to the WHO (2022), universal healthcare coverage should be a core component of health policies for migrants, as their health is closely linked to broader public health and human rights (118). Yet migration is often viewed through a security lens, leading to restrictive policies and practices. Severoni et al. (119) call for a fundamental shift in how universal health coverage is conceptualized — one that embraces shared community responsibility and extends coverage beyond national borders, reflecting the realities of our increasingly interconnected world.

Several countries have demonstrated that rights-based, inclusive HIV programs for migrants are both feasible and effective. Thailand’s Migrant Health Insurance Card Scheme and One Stop Service Registration extend free universal healthcare, including HIV services, to all migrants regardless of documentation status, offering a model of equitable policy, even though bureaucratic delays, fear of arrest, and poor inter-ministerial coordination still limit enrollment (54). In Spain, legislative reforms initiated in 2018 guaranteed undocumented migrants with proof of residency for 3 months access to the national health system, including HIV care, while community health centers partner with NGOs to provide culturally adapted counselling and peer navigation for African migrants (108, 109). In May 2025, Spain approved legislation to legalize residency and work permits for up to 900,000 migrants over three years (120).

South Africa’s National Health Insurance Fund will not provide ARVs to undocumented migrants living with HIV based on the following provision: “Asylum seekers and illegal foreigners will be covered for notifiable conditions and emergency medical services” (121). However, HIV is not a “notifiable condition of public health concern” in South Africa (122, 123). In 2019, the government of Botswana expanded the provision of free antiretroviral drugs to all migrants, and this change dramatically reduced poor antenatal outcomes among migrant women and narrowed the gap with their local counterparts (124). In France, the Urgent and Vital Care (UVC) program provides essential health services to undocumented migrants who are ineligible for other forms of assistance or insurance. A retrospective cohort study comparing two health care programs — UVC, which offers limited emergency care, and the State Medical Aid (SMA), which provides more comprehensive coverage — found that patients in the UVC program had longer hospital stays, higher hospitalization costs, and were more likely to require intensive care than those in the SMA program (125). These findings suggest that providing more comprehensive healthcare access through the SMA program is more cost-effective for the French healthcare system. These initiatives show that integrating legal entitlements with service-level innovations can mitigate the structural barriers that undermine the AAAQ principles.

Integrated service models that combine HIV care with legal aid, psychosocial support, and navigation assistance, which can be seen in NGO-run migrant health centers in Europe and Southern Africa, help migrants assert their health rights while addressing immediate barriers to care (81). Embedding these practices into universal healthcare coverage frameworks ensures sustainability but requires long-term political will, cross-sector coordination, and stable financing. Without institutionalizing these approaches, gains remain vulnerable to policy rollbacks, donor funding cuts, and rising xenophobia. The challenge ahead lies in scaling such inclusive models while safeguarding them against the political and economic shifts that so often erode migrant rights.

**6 A shared responsibility for financing healthcare for migrants**

This strategy addresses availability (ensuring sustained ART, PrEP, and testing programs) and quality (avoiding treatment interruptions due to funding gaps) barriers. It confronts structural violence by challenging exclusionary citizenship-based financing models and shifting responsibility from resource-poor host countries to equitable global pooling mechanisms.

Sustained HIV care for migrants requires coordinated financial support that transcends borders and sectors. The WHO 2022 Report on the Health of Refugees and Migrants called for precisely this paradigm shift, treating migrant health as a shared responsibility and promoting coordination among health ministries, social protection systems, labor sectors, UN agencies, NGOs, and migrant-led organizations. Translating such policy into practice requires multi-sectoral governance structures that bridge migration and health, while acknowledging universal healthcare coverage as a guiding principle (118).

A primary challenge is the prevailing expectation that host countries will bear the full financial burden of migrant health services. This stance is inequitable and unsustainable, particularly for low- and middle-income countries hosting large migrant populations without sufficient capacity to meet the health needs of migrants living with HIV. Regional approaches offer a pragmatic starting point. In the European Union, the European Health Insurance Card (EHIC) allows citizens to access services across member states, with costs reimbursed between the country of origin and destination. Adaptations of such mechanisms could be explored in other regional blocs, including the African Union, the Association of Southeast Asian Nations or the Intergovernmental Authority on Development, where most migration is intra-regional. Yet, the EHIC experience also reveals pitfalls. Uptake remains uneven, and poorer countries face financial strain when costs outpace their health systems’ capacities (126, 127). Without regional risk pooling to offset disparities, these models risk perpetuating inequities rather than alleviating them. This pool could be funded by compulsory, progressive (income-based) contributions from countries in the region, complemented by innovative mechanisms such as earmarked multinational corporate taxes or allocations from climate finance funds to address health needs linked to climate-induced displacement (119, 128). This pool would guarantee a minimum package of essential region-specific healthcare services for migrants, with allocations based on assessed needs and service utilization, while leveraging existing financing and governance structures to avoid duplication.

Rising nationalism, aid retrenchment, and the erosion of multilateralism pose challenges, but the interdependence of health across borders makes solidarity not just desirable but imperative. In an era defined by climate change, protracted conflicts, and economic instability, everyone is a potential migrant. Universal health coverage must therefore be reimagined to transcend borders, be grounded in legal obligations, be implemented through multisectoral partnerships, and be sustained by equitable global financing if the right to health is to be realized for all.

**7 Developing solutions that cater to the specific needs of uniquely situated migrants like women, youth, and lesbian, gay, bisexual, transgender, queer, or questioning (LGBTQI+) individuals**

This strategy strengthens acceptability (services tailored to cultural, gender, and identity needs) and quality (specialized provider competencies). It exposes structural violence at the intersections of sexism, heteronormativity, and racism.

Lokotola et al. (135) highlight the “mismatch between the realities of health systems constraints and the burgeoning needs of migrant populations across Africa”, a challenge that undermines both health equity and the promise of “health for all”: Young, female, and LGBTQI+ African migrants face compounded vulnerabilities in accessing HIV care and remain underrepresented in both research and programming.

For youth migrants, mobility often coincides with critical transitions in education, employment, and sexual identity formation, heightening exposure to HIV while reducing service engagement (81). Legal status and age-based consent laws can restrict access to HIV testing and sexual and reproductive health services, especially for unaccompanied minors or those with uncertain guardianship. Programs seldom address the intersection of migration-related stressors, mental health needs, and HIV prevention for adolescents, despite evidence that culturally adapted, peer-led approaches improve uptake of testing and adherence among migrant youth (54). A scoping review of migrant youth aged 15–24 in 10 countries highlighted the need to prioritize policies, interventions, and research for targeted HIV services for this population due to their heightened risk of exploitation, migration-related trauma, loss of support systems, and stigma in accessing HIV care (129). This review also noted a gap in studies of migrant youth living with HIV (129).

Female African migrants living with HIV often navigate intersecting forms of discrimination rooted in gender, race, and migration status. Studies in Europe and Southern Africa document that migrant women are more likely to experience delayed diagnosis, barriers to antenatal HIV services, and stigma linked to perceptions of HIV being a “foreign” disease (130, 131). Gender-based violence, both pre- and post-migration, further undermines adherence and retention in care, while undocumented status can deter women from seeking timely services due to fear of deportation (62).

For LGBTQI+ migrants, exclusion is not experienced along a single axis but through compounded effects of being queer, a migrant, and living with HIV. Their vulnerabilities are heightened in host country health systems where homophobia, transphobia, xenophobia, and HIV-related stigma intersect (132). This intersectionality means that LGBTQI+ migrants face discrimination not only as migrants navigating hostile immigration regimes, but also as sexual and gender minorities in health systems shaped by heteronormativity and gender binarism (133). Their multiple marginalized identities expose them to overlapping forms of exclusion, where structural barriers increase risks and erode access to HIV care. This may take the form of environmental stressors, such as unemployment, food and housing insecurity, and financial instability, compounded by psychosocial stressors, such as social isolation, abuse, depression, anxiety, and post-traumatic stress disorder (77, 134). A recent systematic review on sexual and gender minority migrants included 20 studies in its final sample. However, this review did not examine how HIV-positive status intersects with sexual and gender minority and migrant identities, likely reflecting the limited research on LGBTQI+ migrants living with HIV (134).

The absence of inclusive, targeted interventions for these populations contravenes the AAAQ principles of availability, accessibility, acceptability, and quality, while also violating states’ obligations under the ICESCR and the UNAIDS/OHCHR International Guidelines on HIV/AIDS and Human Rights. Addressing these overlapping inequities requires host countries to move beyond formal rights protections toward inclusive, rights-based HIV programming that recognizes and responds to the intersectional realities of these uniquely situated migrants living with HIV. It is therefore not simply programmatic good practice — but both a legal obligation and ethical imperative. Furthermore, sexism and heteronormativity are forms of structural violence that compound the challenges faced by African migrants who are also gender and sexual minorities.

## Limitations and strengths

This review synthesized peer-reviewed and grey literature published over the past 20 years, applying the AAAQ human rights framework, integrated with a structural violence lens. Several limitations should be acknowledged. First, although we report our search terms, we did not provide counts of the number of articles screened, retrieved, or excluded. This could introduce bias by omitting relevant publications. However, our hermeneutic approach deliberately emphasized iterative cycles of searching and interpretation, thereby minimizing this risk and allowing us to capture a broad range of perspectives. Narrative reviews, by design, recognize that not all relevant literature may be included. Second, as with any narrative review, the findings are shaped by the authors’ interpretivist lens. While reflexivity can enrich analysis, it also introduces the possibility of selective framing and interpretation of evidence. However, our multidisciplinary team brought diverse expertise and lived experience to the iterative selection and interpretation of evidence, enhancing the rigor and breadth of our synthesis. Third, we limited our review to English-language studies, which may have introduced bias. Fourth, much of the available literature focuses on specific sub-populations or geographic settings, which limits the generalizability of our synthesis across the diverse experiences of African migrants living with HIV. We also recognize that in the current global context, voluntary and forced migration frequently overlap and are difficult to disentangle. Finally, the quality and availability of grey literature are uneven. Nonetheless, such sources are essential for capturing real-world policy and practice perspectives that are often absent in peer-reviewed research.

Despite these limitations, our hermeneutic narrative approach offers several strengths. Iterative cycles of searching, analysis, and interpretation allowed us to engage flexibly and deeply with the literature, capturing insights that systematic or scoping reviews, constrained by rigid protocols, may overlook. Our broad and inclusive search strategy drew on empirical research, systematic reviews, grey literature, and screening reference lists of published articles to ensure comprehensive coverage. A further strength lies in our integration of the AAAQ human rights framework and structural violence lens, which enabled us to move beyond health-related issues to broader questions around racism, capitalism, colonial legacies, and history, among others. This combined framework adds analytical depth and represents a novel contribution to existing literature. By bridging peer-reviewed and grey literature, historical and contemporary sources and multidisciplinary perspectives, this review offers a critical appraisal of the field and actionable insights for policy and practice.

## Conclusion

This narrative review highlights the stark and enduring health inequities faced by African international migrants in accessing HIV care. These inequities are not merely logistical but are rooted in structural injustice and violations of fundamental human rights. From a public health ethics perspective, exclusionary health policies that deny migrants access to HIV care represent avoidable, state-produced harms that contravene duties of justice, equity, and solidarity, and undermine the ethical foundations of universal health coverage. Global goals of “leaving no one behind” will remain hollow promises unless we directly confront the legal, structural, and rights-based barriers that restrict migrants’ access to HIV care and the broader social determinants of health. Healthcare is not a privilege; it is an inalienable human right. Infringements on availability, accessibility, acceptability, quality, and equality are not just policy failures, but constitute breaches of international legal obligations and commitments under the ICESCR, General Comment 14, and UNAIDS/OHCHR guidelines.

Global responses have moved at a snail’s pace. The time for half measures has long passed. Urgent and decisive action is needed to craft inclusive, person-centered health policies and service delivery models for migrants. Yet, as needs grow more pressing, the global infrastructure designed to support them is under siege. The draconian termination of USAID, declining support for UNAIDS, WHO, and other multilateral institutions, and the rising tide of anti-migrant rhetoric globally threaten the capacity of systems that serve migrants living with HIV. These shifts risk further undermining efforts to address upstream determinants of health and access to care.

Reversing this trajectory requires bold reforms in how HIV services are conceptualized, financed, and delivered in migrant-hosting contexts. Inclusive health laws, migrant-competent provider training, robust surveillance, community involvement, and cross-border treatment continuity must become core pillars of a rights-based response. Equity in access must be the norm, not the exception. The COVID-19 pandemic demonstrated that rapid structural change is possible when political will is mobilized; the same urgency is needed now. Our proposed strategies provide a roadmap for transforming decades of stalled progress into meaningful action. Rights-based approaches are not aspirational; they are indispensable.
